# iTRAQ proteomics of sentinel lymph nodes for identification of extracellular matrix proteins to flag metastasis in early breast cancer

**DOI:** 10.1038/s41598-022-12352-9

**Published:** 2022-05-22

**Authors:** Sheetal Pathania, Mohd Imran Khan, Sabyasachi Bandyopadhyay, Suneet Shekhar Singh, Komal Rani, Tanvi Ramesh Parashar, Jnaneshwari Jayaram, Piyush Ranjan Mishra, Anurag Srivastava, Sandeep Mathur, Smriti Hari, Perumal Vanamail, Gururao Hariprasad

**Affiliations:** 1 Department of Biophysics, All India Institute of Medical Sciences, Ansari nagar, New Delhi, 110029 India; 2 Proteomics Lab, Centralized Core Research Facility, All India Institute of Medical Sciences, New Delhi, 110029 India; 3 Department of Surgery, All India Institute of Medical Sciences, Ansari nagar, New Delhi, 110029 India; 4 Department of Pathology, All India Institute of Medical Sciences, Ansari nagar, New Delhi, 110029 India; 5 Department of Radiology, All India Institute of Medical Sciences, Ansari nagar, New Delhi, 110029 India; 6 Department of Obstetrics and Gynaecology, All India Institute of Medical Sciences, Ansari nagar, New Delhi, 110029 India

**Keywords:** Cancer, Biomarkers

## Abstract

Patients with early breast cancer are affected by metastasis to axillary lymph nodes. Metastasis to these nodes is crucial for staging and quality of surgery. Sentinel Lymph Node Biopsy that is currently used to assess lymph node metastasis is not effective. This necessitates identification of biomarkers that can flag metastasis. Early stage breast cancer patients were recruited. Surgical resection of breast was followed by identification of sentinel lymph nodes. Fresh frozen section biopsy was used to assign metastatic and non-metastatic sentinel lymph nodes. Discovery phase included iTRAQ proteomics coupled with mass spectrometric analysis to identify differentially expressed proteins. Data is available via ProteomeXchange with identifier **PXD027668**. Validation was done by bioinformatic analysis and ELISA. There were 2398 unique protein groups and 109 differentially expressed proteins comparing metastatic and non-metastatic lymph nodes. Forty nine proteins were up-regulated, and sixty proteins that were down regulated in metastatic group. Bioinformatic analysis showed ECM-receptor interaction pathways to be implicated in lymph node metastasis. ELISA confirmed up-regulation of ECM proteins in metastatic lymph nodes. ECM proteins have requisite parameters to be developed as a diagnostic tool to assess status of sentinel lymph nodes to guide surgical intervention in early breast cancer.

## Introduction

Breast cancer is a common malignant disease among women population worldwide and is characterized by the highest cancer incidence, high recurrence rate, morbidity, mortality and poor prognosis^1^. Globally breast cancer accounted for 11.7% of the estimated 19.3 million new cancer cases in 2020 in both the sexes and about 24.5% of all cancer cases among women^2^. The International agency for cancer research has reported that breast cancer ranks number one cancer worldwide with an incidence of 22.5 lakh and a mortality rate of nearly seven lakh^3^. The latest cancer registry of Indian Council of Medical Research has reported that breast cancer ranks number one cancer in India with an incidence rate of 25.8 per lakh, and a mortality rate of 12.7per lakh^4^. The breast cancer incidence cases are expected to increase by more than 46% by 2040^5^. As per the cancer death cause analysis, it is not the primary breast tumor but metastasis to distant organs that is responsible for the death of over 90% of breast cancer patients^6^. Breast cancer accounts for 11.6% of distant malignancies in both men and women globally^7^.

The TNM (tumor, node, metastasis) classification is a globally recognised standard to assign the stages for breast cancer. Stage 0 the non-invasive; stage I, IIA, IIB, IIIA describes early breast cancer which is confined to the breast with or without axillary lymph node involvement; stage IIIB, IIIC describes locally advanced and, stage IV describes breast cancer that has spread to other parts of the body like liver, lungs, brain and bones termed as metastatic breast cancer^8,9^.

Axillary lymph nodes represent the main stay of lymphatic drainage from the breast, and the first lymph node among them to be affected is called the sentinel lymph node^10^. Sentinel lymph node biopsy (SLNB) is the standard diagnostic procedure for patients with early-stage breast cancer to assess the status of lymph node^11^. Identification of this node is made by injecting radioactive tracer into the breast in the vicinity of the tumour, followed by its excision, and a biopsy that reflects the histological characteristics and status of nodes in the axilla^12,13^. Surgeons carry out axillary lymph node dissection (ALND) in breast cancer for cases where SLNB is positive and abandon this intervention if it is negative^14,15^. Identification and assessment of sentinel lymph node metastasis dictates the right surgical approach adopted and the prognosis in patients with early breast cancer^16^.

Recently, potential biomarkers have been identified to understand lymph node metastasis in early breast cancer^17–20^. Unfortunately, each of these have poor sensitivity and specificity, and has therefore not been translated into a diagnostic tool as desired by the surgeon. A number of imaging techniques too have been used to pick up axillary lymph node metastasis, but these techniques are neither efficient, nor applicable during the surgical procedures^21–26^.

Our team has been identifying potential protein biomarkers in various clinical phenotypes in the last few years^27,28^. In the recent past, we have carried out a gel based proteomics experiment to identify biomarkers that can flag lymph node metastasis in early breast cancer^29^. In this study, we seek to carry out an isobaric label based proteomic experiment to delineate protein signatures that can accurately reflect the metastatic state of the sentinel lymph nodes in early breast cancer.

## Methodology

### Ethics and patient recruitment

This study was conducted after approval was obtained from the Institute Ethics Committee at All India Institute of Medical Sciences New Delhi, India (Ref. No. IECPG- 27/23.01.2019, RT-03/28.02.2019). The procedures were followed as per the ethical standards formulated in the Helinski declaration. Early breast Cancer patients were screened and admitted at the Department of Surgery. Detailed information on the study was explained to the recruited patients and informed consent was obtained from them. Bedside examination included clinical history, symptoms, signs and general examination. Patients suspected of having early breast cancer were subjected to mammography imaging. As explained later in this section, sentinel lymph node biopsy tissues from these patients were sent to Department of Pathology for histopathology, which was the gold standard for assigning the clinical phenotypes of sentinel lymph node metastasis (SLNM +) and sentinel lymph node without metastasis (SLNM-). For the discovery phase of the proteomic experiment, we took 5 patients with SLNB + , 5 patients with SLNB- and, two benign breast tumor tissues as cancer controls. For the validation phase of experiment, we took 13 patients each with SLNM + and SLNM-.

### Patient inclusion and exclusion criteria

Staging of the breast cancer were determined as per to the American Joint Committee on Cancer (AJCC) cancer staging criteria. Inclusion and exclusion criteria were used just as in our previous study^29^. Women with early invasive intra-ductal breast cancer as per the WHO classification of the tumor, and who had not undergone any therapeutic intervention were recruited into the study. Patients with advanced breast cancer, and patients with early breast cancer who had either received chemotherapy or radiotherapy were excluded from the study.

### Identification and excision of sentinel lymph node

Technetium tagged sulphur colloid was injected intra-dermally into the lower inner quadrant of the affected breast, two hours prior to the surgery^29^. In operation theatre, 1 ml of 1% fluorescent methylene blue dye diluted in 4 ml saline was injected at multiple sites intra-dermally around areolar region and in sub-areolar region. After five minutes of gentle massage, an incision was made on axillary skin crease at the site of maximum radioactivity. By using blunt and sharp dissection, methylene fluorescent lymphatics was identified using a blue light lamp, and blue lymphatics was identified by direct visualization^30^. Lymph node having highest count was considered as sentinel node and was excised.

### Sentinel lymph node tissue sample collection

After the excision of sentinel lymph node, adherent fat tissues were neatly removed, and blood stains were washed thoroughly with 1X PBS (pH 7.4)^29^. The nodes were longitudinally sectioned to obtain 5 mm thick slices. One set of alternate slices was sent to the Department of Pathology for histopathological assessment. The other set of slices were taken to proteomics facility and stored at − 80 °C for iTRAQ based proteomic experiments.

### Histopathology

Histopathological procedures that were followed were those standardized in our previous study^29^. Lymph node slices were fixed into formalin fixed paraffin embedded blocks. These were further sectioned into 4 μm poly-L-lysine-coated slides. These paraffin sections were deparaffinised with three subsequent washes in xylene and then rehydrated by washing them stepwise in 100% ethanol, 90% ethanol, 70% ethanol and distilled water. The sections were stained with hematoxylin and washed in running water for 5 min. The slides were then stained in eosin solution for two minutes and then rinsed with 95% ethanol. The slides were then subjected to 100% ethanol for two minutes, twice. After final exposure to xylene, a drop of Distyrene Plasticizer Xylene (DPX) was used to mount the tissue on each slide and covered with a glass cover-slip. The slides were examined under the microscope and sentinel lymph node tissue samples were annotated as either Sentinel Lymph Node Metastatic (SLNM +), or Sentinel Lymph Node Non-Metastatic (SLNM-).

### Mammography

All recruited patients in the study underwent full-field digital mammography in cranio-caudal projection and medio-lateral oblique projection. The effective dose of a four view mammogram ranged from 4 to 6 mega gray. The evaluation of mammogram was done according to the Breast Imaging Reporting and Data System classification (BIRADS).

### Sample phenotyping and protein isolation

Phenotyping and protein isolation was done using protocols standardized in our previous study^29^. The sentinel lymph node tissue sections which were stored at − 80 °C were annotated as either SLNM + or SLNM- based on histopathology finding. The tissue samples were minced and the proteins were solubilized in 120 μl of lysis buffer that contained 8 M urea, 2 M thiourea and 4% 3-[(3-Cholamidopropyl)dimethylammonio]1-propanesulfonate (CHAPS). The tissue was homogenized by sonication at an interval of 3 s and vortexed for 2 min. The samples were then centrifuged at 15,000 rpm for 20 min at 4 °C, debris was discarded and the supernatant was transferred onto a fresh eppendorf tube. Protein extracted with lysis solution was buffer exchanged with 100 mM Triethylammonium bicarbonate (TEAB) using a 3 kDa cut off membrane filters to bring down the concentration of urea well below 0.1 M. Protein amount was quantified using the Bradford assay using 1 μg/μl of Bovine serum albumin as a standard.

### Isobaric tags for relative and absolute quantitation (iTRAQ) labelling

**Figure 1:**
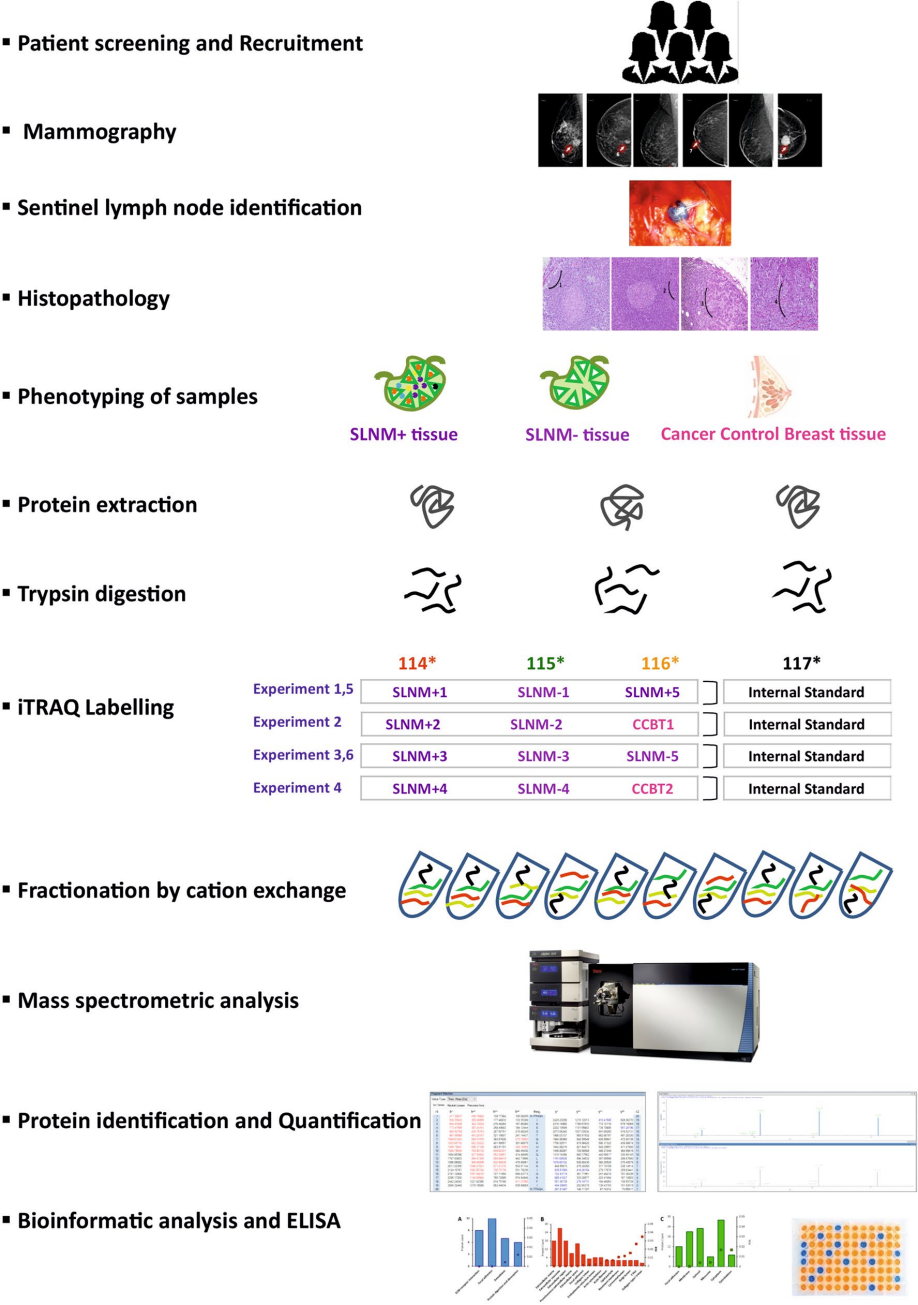
Flowchart depicting the methodology used in the study.

Five sets of SLNM + tissue samples, five sets of SLNM- tissue samples and two cancer control benign breast tumor tissues were taken for iTRAQ experiment. The design of iTRAQ experiment is illustrated in Fig. 1. Each experiment composed of at least one SLNM + , one SLNM- and, one of either of these two phenotypes or a cancer control benign breast tumor tissue. The equimolar culmination of the three phenotypes was made into an internal standard for the sake of normalization for each of the four experiments^27^. 80 μg of protein from each phenotype sample was reduced with 25 mM DTT for 30 min at 60 °C and alkylated with 55 mM iodoacetamide for 20 min at room temperature. Each of these proteins samples were digested for 16 h with trypsin in 1:10 ratio at 37 °C. Digested peptides were then labelled with iTRAQ 4-plex reagents, 114 (sentinel lymph node metastasis), 115 (sentinel lymph node without metastasis, 116 (one of either of these two phenotypes or cancer control benign breast tumor tissues), and 117 (internal standard) following the protocol provided by manufacturer (AB Sciex, Foster city, CA, USA). In brief, all vials of iTRAQ labelling tags were reconstituted in 70 μl of absolute ethanol. This was then added to each sample and incubated for 2 h at room temperature, and the reaction was quenched using 50 μl mili-Q water. iTRAQ labelled samples in each experiment were then pooled into a single vial and dried using speedvac. These samples were reconstituted in 8 mM ammonium formate buffer (pH: 3) and were fractionated by cation exchange using isotope coded affinity tag cartridge. Peptides were then eluted with 500 μl of gradient elution with 5 mM to 500 mM concentrations range of ammonium formate (pH: 3) to obtain a total of eleven fractions from each experiment. These 44 fractions from the four experiments were vaccum dried and taken for mass spectrometry analysis.

### Mass spectrometry data acquisition

In-house protocols were used for Mass spectrometry data acquisition^27^. Peptides from the 44 fractions were desalted and concentrated using reversed phase ZipTip, and reconstituted in 0.1% formic acid. The peptide fractions were loaded onto analytical column (Acclaim PepMap RSLC C18, 2 μm, 100 Å, 50 μm x 15 cm; Thermo Scientific, Rockford, USA) associated with trap column (Acclaim PepMap C18, 3 μm, 100 Å, 75 μm x 2 cm; Thermo Scientific, Rockford, USA). The peptide separation were performed using EASY-nLC 1200 which was coupled with Orbitrap Fusion Tribrid Mass Spectrometer (Thermo Scientific, Rockford, USA) for Mass spectrometry analysis. The peptide fractions were premixed in loading buffer (Mobile phase A: 100% water and 0.1% formic acid) and 1 μg were loaded on a trap column with a flow rate of 300 nl/min. The retained peptides were washed iso-cractically by loading buffer for 45 min to remove excess salt. The peptides were then resolved on an analytical column with a multi step linear gradient of loading buffer and elution buffer (mobile phase B: 80% acetonitrile and 0.1% formic acid) at a flow rate of 250 nl/min. The gradient elution were initiated using 5% elution buffer and were held for 1 min, with linear increase rate of 10% for 10 min, 35% for 70 min and 50% for 80 min. The gradient elutions were held at 80% mobile phase B for 8 min before being re-equilibrated to 5% mobile phase B for 18 min. The mass spectrometer was operated in data dependent acquisition (DDA) mode. The full MS spectra was acquired in positive ion mode in m/z ratio of 350–2000 Da, with a 100 milli second MS accumulation time, whereas the MS/MS product ion scans were performed in the mass range of 100–2000 Da with a 7 milli second accumulation time in Orbitrap mass analyzer. The mass spectrometric setting included Ion spray voltage floating of 1900 V. For 3 s former target was excluded and 25 ions were monitored per MS cycle. DDA advanced ‘rolling collision energy’ were applied for subsequent MS/MS scans with normalized High energy Collision induced Dissociation (HCD) collision energy set to 35%.

### Data analysis and reporter ion quantification

Raw files from the Orbitrap Fusion Mass Spectrometer were processed using Proteome Discoverer (version 2.4.1.15) analysis software^31^. Both MS and MS/MS spectra were searched using Sequest HT algorithm against a combined UniProt Human proteome database appended to a list of common contaminants provided by Thermo Scientific. Sequest HT parameters were specified as trypsin enzyme, two missed cleavages allowed, minimum peptide length of 6, precursor mass tolerance of 10 ppm and a fragment mass tolerance of 0.05 Daltons. The static modification was set to carbamidomethylation (+ 57.021 Da) of cysteine. The dynamic modifications on peptide terminus were set to methionine oxidation (+ 15.995 Da) and iTRAQ 4-plex (+ 144.102 Da) modification, on N-terminus and Lysine (K) residues. Since iTRAQ modification was used as dynamic modification, the unmodified or unlabelled peptides and associated proteins were removed from the analysis. Also, dynamic modification was assigned for acetylation (+ 42.011 Da) of protein’s N-terminus. Peptide spectral match (PSM) error rates were determined using the target-decoy strategy coupled to Percolator PSM validation node to trigger the positive and false matches. In Percolator node, the false discovery rate (FDR) was calculated based on the *q*-values of Decoy database search. Data were filtered at the peptide spectral match-level using a strict FDR cut off of 0.01 and relaxed FDR cut off of 0.05 as determined by Percolator. Contaminant and decoy proteins were removed from all data sets prior to downstream analysis. Reporter ion values were calculated on “Reporter Ions Quantifier” node using FTMS mass analyzer setting and HCD activation process. Reporter ions were quantified from MS/MS scans using an integration tolerance of 20 ppm with the most confident centroid setting. The following settings were used to obtain the quantification results: the protein ratio type was the ‘weighted’ geometric mean, normalization with summed intensities and outlier removal was ‘automatic’. The peptide threshold was ‘at least homology’ where peptide score does not exceed absolute threshold but is an outlier from the quasi-normal distribution of random scores. Minimum of two unique peptides were required to be the top ranking matches. In Consensus workflow in “Reporter Ion Quantifier” node, the following settings were applied to increase the quantification accuracy of the analyzed proteins: (i) only unique peptides were used for protein quantification, (ii) precursor co-isolation threshold was considered 25%, (iii) average reporter signal to noise ratio threshold was considered 10 and, (iv) peptide normalization was done with respect to the total peptide amount. At the level of protein analysis, further normalization was done where the protein abundance of individual sample was scaled by the abundance of the internal standard which was labelled with channel 117 of iTRAQ 4-plex reagent. On obtaining the results, multiple filter criteria were applied and only those proteins were considered for differential expression analysis which had: (i) FDR confidence threshold as medium during identification by Sequest-HT, (ii) presence of atleast two unique peptides and, (iii) peak found at found in sample.

Expression fold change ratio of ≥ 1.5 and ≤ 0.66 were considered as up-regulated and down-regulated proteins. Proteins with fold change ration between 1.5 and 0.66 were considered as house-keeping proteins. A multiple students *t*-test was applied to the whole set of differentially expressed proteins and a volcano plot with a *p*-value < 0.05 was generated to graphically represent the up-regulated, down-regulated and house-keeping proteins. Those proteins that had a consistent expression pattern in atleast four of the six experiments were considered to be potential biomarker candidates to differentiate SLNM + and SLNM-. The relative ratios of protein abundances of only the up-regulated proteins was compared between metastatic group and cancer control breast tissues was used to estimate the possible tissue source.

### KEGG pathway and Gene Ontology analysis

Differentially expressed genes were imported on DAVID^32^ (Database for Annotation, Visualization and Integrated Discovery, version 6.8, https://david.ncifcrf.gov/tools.jsp) functional annotation tool and Functional Enrichment analysis was done. Homo Sapiens was used as background species and the enrichment analysis was run for Cellular Component in Gene Ontology (GO_CC) and Kyoto Encyclopaedia of Genes and Genomes (KEGG) pathways. Only those results that had FDR adjusted *p*-values ≤ 0.05 were considered.

### ELISA

Twelve differentialy expressed proteins: α-crystallin B chain, monoamine oxidase, caveolin-1, collagen α-1, desmin, fibrillin-1, long-chain-fatty-acid–coA ligase 1, laminin subunit α-4, heterogeneous nuclear ribonucleoprotein D, non-histone chromosomal protein, cathelicidin antimicrobial peptide, rho GDP-dissociation inhibitor 2 were chosen for validation phase of the experiment. The protein concentrations of these proteins were quantified in validation set of 13 SLNM + and 12SLNM- patients using ELISA according to the manufacturer's instructions (Bioassay Technology Laboratory, Shanghai, China). All determinations were performed in duplicates according to the manufacturer’s recommendations. Differences between SLNM + and SLNM- groups of patients were calculated using independent Student t-test; values of *p* < 0.05 were considered significant.

### Statistical analysis

Normalization of the proteins was done using MetaboAnalyst (version 5.0) software (https://www.metaboanalyst.ca/MetaboAnalyst/ModuleView.xhtml)^33^ using sum of protein abundances. Data transoformation using generalized logirithm and has been scaled using Pareto scaling option. Data analysis for ELISA was carried out using STATA version 16.0 version. Protein concentrations that could be used as cut-off to distinguish between metastatic state and non-metastatic state, were estimated based on Receiver Operating Characteristics (ROC) analysis of the ELISA data. The non-parameteric ROC analysis was carried out using DeLong method. Area under the curve (AUC) was obtained with 95% confidence limits. Optimum cut-off value was obtained at which Yuden Index (sensitivity + specificity-1) was maximum. Percentage correct classification and likelihood ratio values were computed. Statistical significance level *P* < 0.05 was adopted to test the significance of the AUC.

### Ethics approval and consent to participate

This study was conducted after approval was obtained from the Institute Ethics Committee at All India Institute of Medical Sciences New Delhi, India (Ref. No. IECPG- 27/23.01.2019, RT-03/28.02.2019).

## Result

### Clinical profile

**Figure 2:**
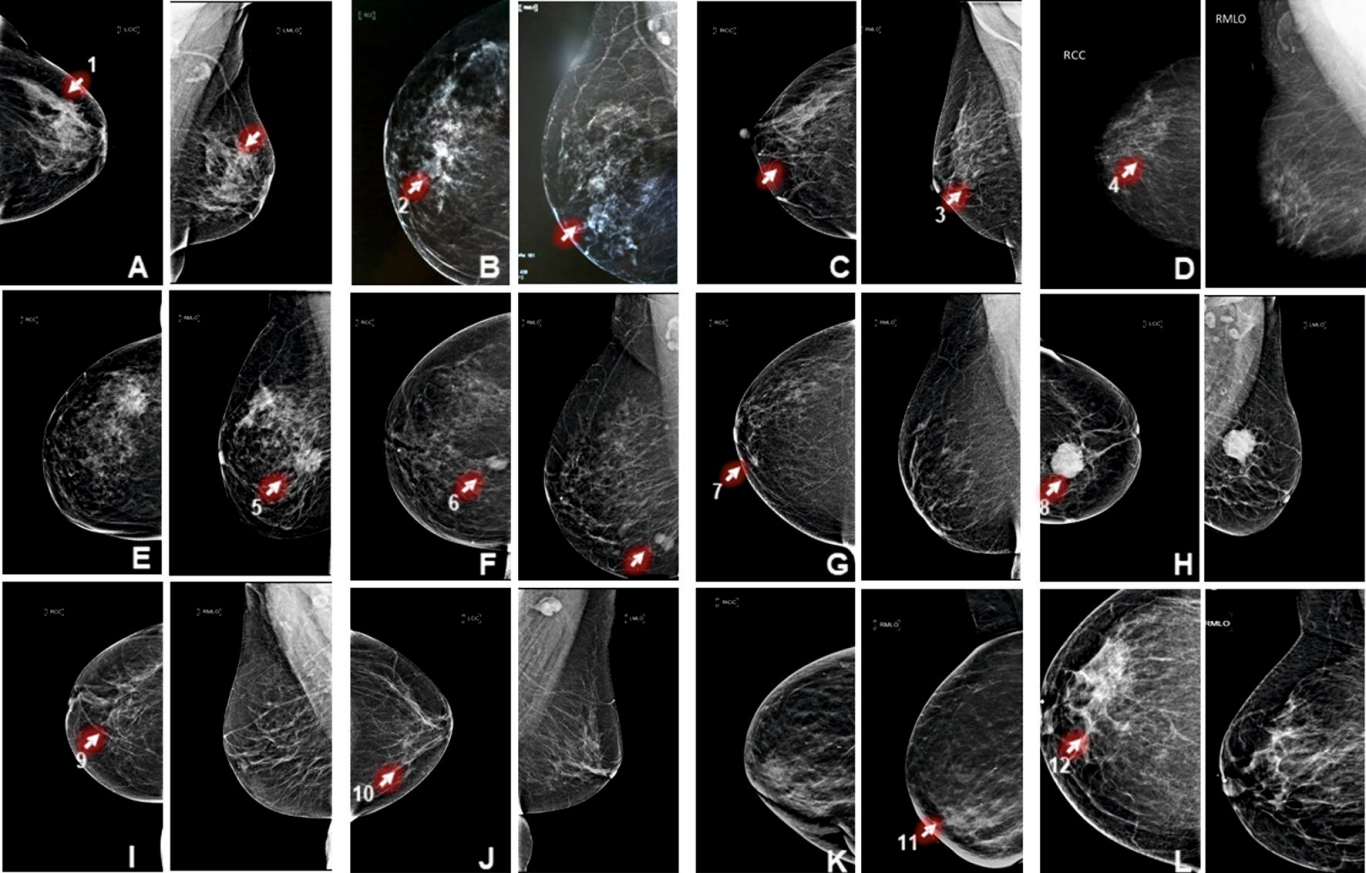
Medio-lateral oblique and cranio-caudal mammography images of patients recruited in the discovery phase of the study. (**A**)–(**J**) shows features of early invasive breast cancer and K-L shows features of benign breast tumor. (1) focal architectural disruption; (2) speculated mass; (3) micro-calcification; (4) micro-calcification along with subtle focal asymmetry; (5) multiple speculated masses; (6) macro-lobulated mass; (7) subtle area of focal architectural distortion; (8) circumscribed mass with focal speculation; (9) architectural distortion; (10) unilateral axillary lymphadenopathy; (11) well circumscribed masses; (12) fat containing masses.

**Figure 3:**
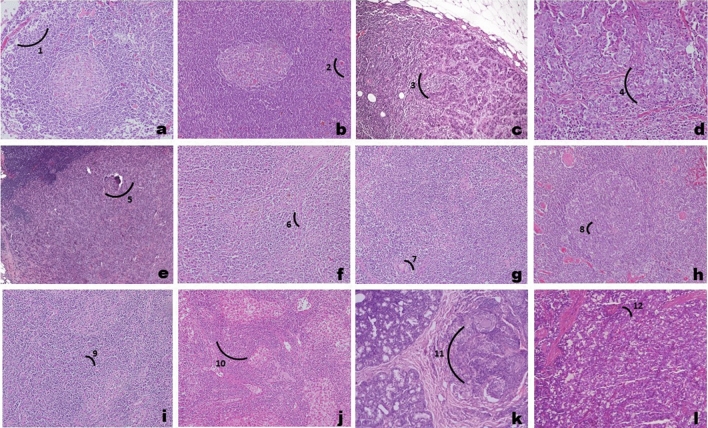
Photomicrographs showing sentinel lymph node with metastasis (**a**–**e**), sentinel lymph node without metastasis (**f**–**j**), and benign breast tumor tissue (**k**,**l**) in hematoxylin and eosin stained sections under light microscopy. (1) complete effacement of architecture by a metastatic cancer cells; (2) infilteration by a metastatic cancer cells; (3) presence of tumor cell nests in the sub-capsular area with a part of the lymph node seen at one edge; (4) presence of large area of tumor cell nest replacing the normal lymph node parenchyma; (5) complete effacement of architecture by metastatic cancer cells. (6–10) shows prominent reactive centre without any evidence of tumor cells in the lymph node parenchyma and depicts paracortical expansion with vascular proliferation indicative of reactive lymphadenopathy. (11–12) depicts benign breast lesion consisting of terminal duct lobular units with bi-layered epithelium and a slant inter venium stroma.

Based on the inclusion and exclusion criteria a total of twenty eight patients with early breast cancer were recruited to this study. From among these, thirteen patients were those who had sentinel lymph node metastasis; thirteen patients were those who did not have sentinel lymph node metastasis, and two patients who were diagnosed with fibroadenoma were recruited to procure breast tissue that would serve as cancer controls. From among these, five with SLNM + , five with SLNM-, and the two cancer control benign breast tumor were chosen for the discovery phase of proteomic experiments by iTRAQ. The clinical profile of these twelve patients is provided in Table 1. All patients with early breast cancer were confirmed by mammography and sentinel lymph node tissue samples annotation either with sentinel lymph node metastasis or without metastasis were done using histopathology evaluation. The mammography images of patients who were considered for the discovery phase of the study are shown in Fig. 2. The assessment of mammogram was performed according to scores of BIRADS (Breast Imaging Reporting and Data System) classification. The hematoxylin and eosin stained slides for the five metastatic, five non-metastatic sentinel lymph nodes and two cancer control benign breast tumor tissues are shown in Fig. 3.

**Table 1:** Clinical profile of patients recruited in the discovery phase of the iTRAQ experiment.

S. No	Patient id	Age (yrs)	Mammography	Size(cm)	ER/PR/Her2	Histopathology	Phenotype
1	KD42	42	Early breast cancer	2.5 × 2 × 1.8	+ , + , −	Complete effacement of architecture	SLNM +
2	UG51	51	Early breast cancer	5.2 × 1.8 × 1.4	+ , −, +	Infiltration by a metastatic cancer cells	SLNM +
3	PK47	47	Early breast cancer	3.5 × 2 × 1	+ , −, −	Tumor cell nests in the sub-capsular area	SLNM +
4	KM50	50	Early breast cancer	2.5 × 1.5 × 1.5	+ , + , −	Tumor cell nest replacing the normal lymph node parenchyma	SLNM +
5	SP52	52	Early breast cancer	3 × 2.5 × 2	+ , + ,-	Tumor cell invading into stroma	SLNM +
6	PD54	54	Early breast cancer	3.5 × 3.1 × 2.5	−, −, −	Reactive lymphadenopathy without any evidence of tumor cells	SLNM−
7	MK54	54	Early breast cancer	1.0 × 0.8 × 0.5	+ , −, −	Reactive lymphadenopathy without any evidence of tumor cells	SLNM−
8	LW52	52	Early breast cancer	3.3 × 2.5 × 1.5	+ , −, +	Reactive lymphadenopathy without any evidence of tumor cells	SLNM−
9	SM59	59	Early breast cancer	3 × 3 × 2.5	−, −, −	Reactive lymphadenopathy without any evidence of tumor cells	SLNM-
10	CD	47	Early breast cancer	1.5 × 1.5 × 5	+ , −, −	Reactive lymphadenopathy without any evidence of tumor cells	SLNM-
11	SC36	36	Well circumscribed mass	4.3 × 3 × 2.5	−, −, −	Terminal duct lobular units with bilayered epithelium and a slant interveniumstroma	Benign breast tumor
12	SS59	59	Fat containing mass	3 × 3 × 2.5	+ , + , −	Terminal duct lobular units with bilayered epithelium and a slant interveniumstroma	Benign breast tumor

### iTRAQ labelling

In our study, proteins isolated from metastatic and non-metastatic sentinel lymph node tissues, and cancer control benign breast tumor tissues were labelled with isobaric tags 114, 115 and 116. Equimolar protein concentrations of proteins from each representative phenotype in the experiment were labelled with tag 117 for the sake of normalization. Out of 276,572 Peptide Spectrum Matches (PSMs), 269,279 (97.4%) are iTRAQ modified and out of a total of 42,105 peptides, 40,074 peptides (95.2%) were found to be iTRAQ labelled reflective of the labelling efficiency.

### Protein expression

**Figure 4:**
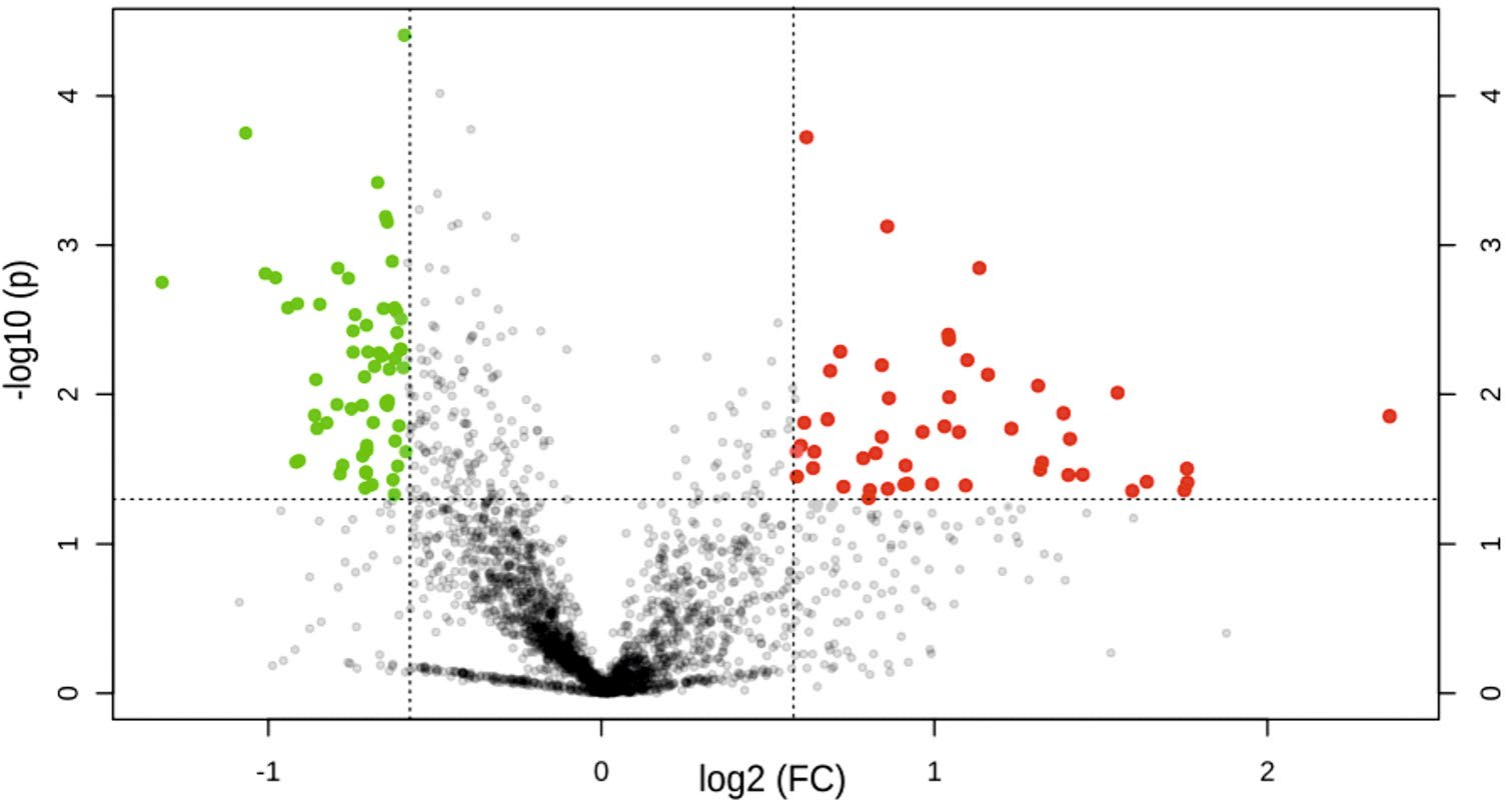
Volcano plot of proteins identified in iTRAQ experiment. Red dots represent up-regulated proteins with > 1.5 fold change, green dots represent down-regulated proteins with < 0.66 fold change. House-keeping proteins with expression fold between 1.5 and 0.66 is shown as greydots. FC indicates Fold Change.

From the 44 fractionated iTRAQ four plex labelled samples, 6335 unique protein groups were identified and after applying stringent filter criteria 2398 proteins were taken up for further analysis. Distribution of these proteins is shown by a normal curve indicative of the quality of the normalization (Supplementary Fig. 1). This process has adjusted for differences among different samples, data transformation and scaling to make individual protein expressions comparable across metastatic and non-metastatic lymph node groups. A pair-wise multiple Students’s *t*-test that incorporates p-value was used to arrive at 109 differentially expressed proteins between SLNM + and SLNM- of which 49 proteins are up-regulated 60 proteins are down-regulated as shown in volcano plot (Fig. 4). The relative abundance ratios of only the up-regulated proteins were compared between metastatic group and cancer control breast tissues. Proteins such as desmin, fibrillin 1, tau-tubulin kinase, transgelin, calponin 1 and myosin 11 that had a ratio of more than one are ones that are native to the lymph node, and proteins such as heat shock protein 6, α-crystalline B, amine oxidase 3, caveolin 1, collagen α1, fibrinogen gamma chain, GAPDH, long chain fatty acid Co-A ligase, laminin subunit α4, membrane primary oxidase, microfibril-associated glycoprotein 4, perilipin 1 and redox regulatory protein that had a ratio of less than one are derived from breast tissue. Based on the functional annotation and their relevance in this study few of these proteins are discussed in Tables 2 and 3.

**Table 2:** Proteins up-regulated in tissue of sentinel lymph node with metastasis as compared to sentinel lymph node without metastasis.

S.No	Protein	Accesion no.	Avg. ratio of SLNM + /SLNM-	Functions	Relevance in our study	References
1	Alpha-crystallin B chain	E9PS12	1.8	Inhibits the autoproteolytic cleavage of caspase-3, interacts with the pro-apoptotic proteins and prevents translocation to mitochondria	Promotes anti-apoptosis	^34,35^
				Inhibits P53 dependent apoptosis by inhibiting Ras activation, blocks UVA cell apoptosis and binds to X-linked inhibitor of apoptosis to inhibit caspase	Promotes anti-apoptosis	^36^
				Associated with transcriptional activation, regulates the Vascular Endothelial Growth Factor (VEGF) and confers VEGF resistance; Induces EGF and anchorage-independent growth through activation of the MEK/ERK pathway	Promotes tumorigenesis	^37^
2	Amine oxidase 3	K7EQZ5	3.4	Regulates myofibroblastic phenotype in a cancer-associated fibroblast	Promotes metastasis	^38,39^
3	Calponin 1	K7ENC5	3.0	Induces cytoskeleton changes that leads to EM	Promotes metastasis and invasion of tumor cell	^40–44^
4	Caveolin 1	C9JKI3	2.2	Regulates intrinsic, extrinsic apoptotic pathway proteins and downstream proteins; Cav-1 induced autophagy alteration influenced apoptosis	Promotes anti-apoptosis	^45,46^
				Influences the levels of EMT (Epithelial to mesenchymal transition) associated markers, transcription factors, MMP and RhoGTPases; Participates in Cav1 dependent trafficking of integrins	Promotes invasion and migration	^47–49^
				Endows anoikis resistance by inactivating caspase-8 and regulates the levels of metastasis-associated proteins like MT4-MMP, MMP9 and TLR4	Promotes metastasis	^50,51^
5	Collagen alpha-1	P02461	5.6	Induces mRNA level of MMP9 and regulates the target gene of Wnt/βcatenin pathways	Promotes invasion	^52,53^
6	Desmin	P17661	2.7	Activates Tumor stromal myofibroblasts and transforming growth factor (TGF)β that switches non-invasive to invasive cancer	Promotes angiogenesis	^54^
7	Fibrillin 1	P35555	3.8	Associates with TGFβ signalling which induces EMT and in turn regulates the expression of E-cadherin, beta-catenin and MMPs	Promotes growth, proliferation and invasion	^55–57^
8	Glyceraldehyde-3-phosphate dehydrogenase	P21695	3.1	Interacts with Sp1 and enhances the expression of SNAIL, a transcriptional inducer of EMT	Promotes invasion and metastasis	^58^
9	Heat shock protein gene 6	K7EP04	2.2	Chaperone focal adhesion kinase, integrin linked kinase and the receptor tyrosine kinases ErbB2 and MET	Promotes metastasis	^59,60^
				Induces epithelial-mesenchymal transition, in which cells switch from a compact shape to a spindle shape	Increases cell motility	^59,61^
10	Laminin subunit alpha 4	Q16363	2.1	Involved in mitogen-activated protein kinases (MAPK) cascades and dual-specificity phosphatases in lamininsignaling in human melanoma cells	Promotes tumor progression, tumor invasion, metastasis	^62,63^
11	Membrane primary amine oxidase	Q16853	3.3	MAOA stabilizes HIF1α, activates the VEGF-A/NRP1 system, and induces expression of TWIST1, an EMT master transcription factor that participates in EMT promotion	Promotes migration, proliferation and invasion	^64^
12	Microfibril-associated glycoprotein 4	K7ES70	2.6	Inhibits Notch1 signaling and interacts with Jagged1	Promotes angiogenesis	^65^
				Interacts with αvβ3 integrin receptor	Promotes tumor cell motility and survival	^66^
13	Myosin-11	P35749	3.0	Link to actin filaments	Promotes breast cancer motility	^67^
				Response to impaired p53, cell adhesion inhibition, protrusion formation	Breast tumor progression	^68^
14	Perilipin-1	O60240	3.0	Regulates lipid network of fatty acid synthase (FASN) and sterol regulatory element-binding protein (SREBP) family	Promotes angiogenesis and metastasis in breast cancer	^69^
15	Redox-regulatory protein FAM213A	Q9BRX8	1.8	miR-211 and FAM213A represes TCF12(transcprition factor 12)	Promotes oncogenesis	^70^
16	Transgelin	K7ERK4	3	Associates with TGFβ/Smad-3	Promotes invasion	^71–73^
				Regulates the production of MMP2	Promotes metastasis	^72,73^

**Table 3:** Proteins down-regulated in tissue of sentinel lymph node metastasis as compared to sentinel lymph node without metastasis.

S. No	Proteins identified	Accession ID	Avg Ratio SLNM + /SLNM-	Functions	Relevance in the study	References
1	Coagulation factor XIII A chain	H0Y796	− 1.5	Coagulation factor Xa inhibits cancer cell migration in a PAR-1 dependent manner	Inhibition of cancer cell migration	^74^
				Causes activation of LIMK1/2 which inactivatescofilin and leads to inhibition of actinide polymerization	Suppresses cancer motility	^74^
2	Heterogeneous nuclear ribonucleoprotein D	D6RBQ9	− 4.0	Inhibits STAT3 and MMP2 via WNT/ TCF4 signalling pathways	Inhibits breast metastasis	^75–77^
				Increases the expression of E-cadherin and down-regulates Twist1 and Snail signalling pathways	Inhibits breast cancer progression	^78^
3	Non-histone chromosomal protein HMG-14	A6NEL0	− 2.0	Transcriptional repression	Acts as tumor-suppressor gene	^79^
4	Histone H1.3	P16402	− 2.1	Inhibits *H19* expression (non codingoncogene) by preferential occupancy at the ICR (imprinting control region) of H19 and regulating DNA methylation at this region	Decreases the growth rate, colony formation in cancer cell and suppression of epithelial carcinogenesis	^80^
5	Cathelicidin antimicrobial peptide	P49913	− 4.7	Inhibitstumor-associated fibroblasts (TAFs) through suppression of epithelial-mesenchymal transition (EMT) and disruption of the cytoskeleton	Suppresses cancer cell proliferation	^81^
6	High mobility group nucleosome-binding domain-containing protein4	O00479	− 2.5	HMG along with (miRNA) downregulates SOX4 expression	Inhibits metastasis	^82^
7	Rho GDP-dissociation inhibitor 2	F5H6Q0	− 1.6	GDI2 inhibits GTPase function by binding to Rac 1	Inhibition of metastasis and cancer growth	^83^
8	Thymosin beta-4	P62328	− 1.7	Down-regulates STAT3–MMP2 signaling	Suppresses migration and invasion	^84,85^

### Validation by bioinformatic analysis and ELISA

**Figure 5:**
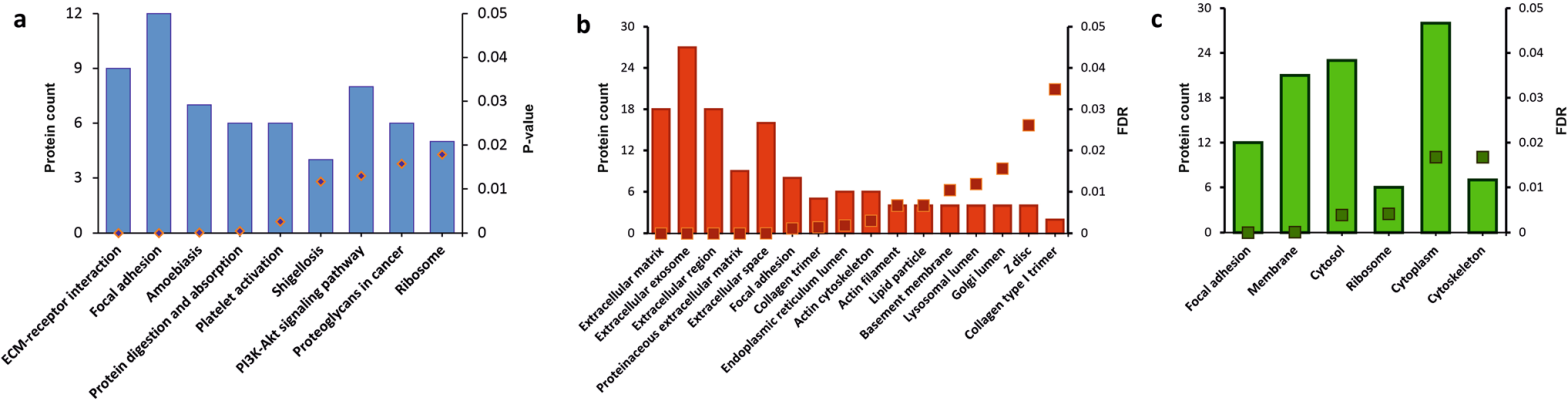
KEGG pathways analysis and Gene Ontology of differentially expressed proteins. (**a**) KEGG pathway analysis showing the various possible pathways regulated by the differentially expressed proteins; (**b**) Gene Ontology for cellular components showing distribution of 49 up-regulated proteins across various cellular organelles; (**c**) Gene Ontology for cellular components showing distribution of 60down-regulated proteins across various cellular organelles. Square boxes designate the FDR corrected p-values.

**Figure 6:**
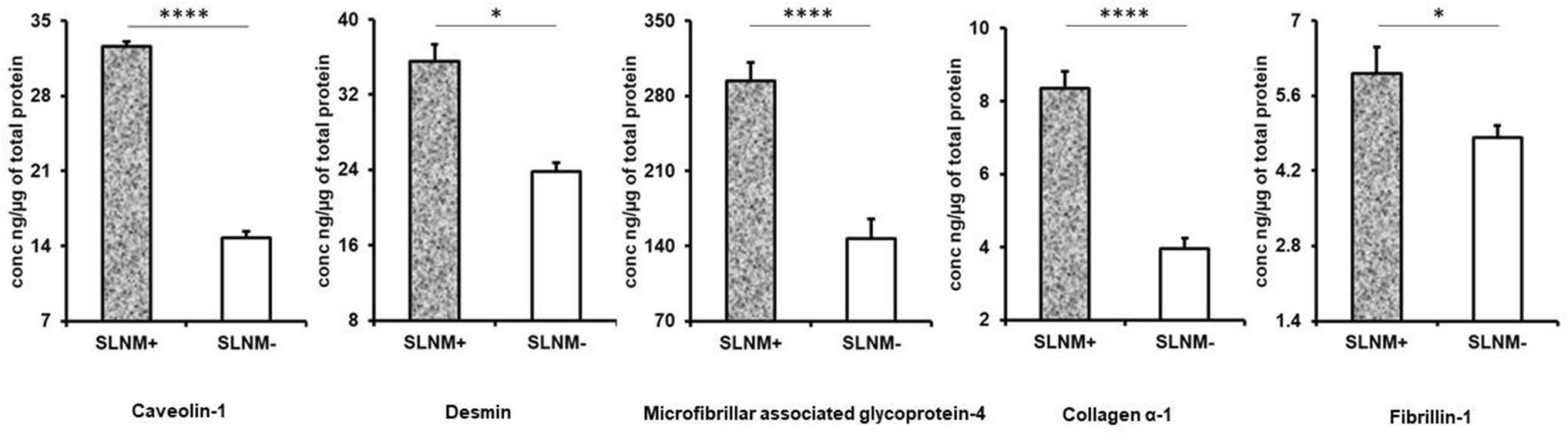
ELISA. Bar diagrams showing the ratio of protein expression of (**a**) caveolin-1, (**b**) desmin, (**c**) microfibrillar associated glycoprotein 4 (**d**) collagen *α *− 1, and (**e**) fibrillin-1 in metastatic patients (black) and non-metastatic patients (grey). *indicates *P* < 0.05; ****indicates *P* < 0.0001.

KEGG pathway analysis highlights the role of the differentially expressed proteins in various pathways (Fig. 5a). The most interesting feature is the ECM-receptor interaction that implicates seven proteins that include various isoforms of collagen-α. Apart from ECM-receptor interaction pathways, focal adhesion and PI3K-Akt signalling feature prominently in the pathway analysis. Gene Ontology for Cellular Component (GO_CC) enrichment that was carried for the 49 upregulated proteins shows that majority of the proteins belong to extra cellular component (Fig. 5b). The next component with highest number of proteins was focal adhesion component. Focal adhesions are large macromolecular assemblies through which mechanical force and regulatory signals are transmitted between the extracellular matrix and an interacting cell. Implication of extracellular proteins is therefore quite evident in breast cancer metastasis. Gene Ontology for Cellular Component (GO_CC) enrichment that was carried for the 61 down-regulated proteins shows majority of the proteins being confined to cytoplasmic, cytosol and membrane component (Fig. 5c). Based on the results of bioinformatic studies, ELISA was performed on upregulated extracellular proteins in SLNM + . Caveolin 1, Desmin, Microfibrillar associated glycoprotein,Collagen α4 and Fibrillin 1 were confirmed to be elevated in SLNM + as compared to SLNM- (Fig. 6) (Supplementary Table 1). These proteins have a minimum of two fold higher expression in SLNM + as compared to SLNM-.

## Discussion

The predominant age group of early breast cancer patient recruited in our study was 36–59 years. Most of the patients presented with lump, nipple discharge, nipple retraction, pain in one part of the breast. Progesterone, esterogen and HER-2 status did not have any correlation with the lymph node status of early breast cancer. Patients clinically diagnosed as early breast cancer were subjected for mammography screening to confirm the type of breast cancer. Twenty five patients were diagnosed with early breast cancer on the basis of their characteristic features like abnormal masses and collection of calcification. As normal control tissues could not be procured due to ethical concerns, Two fibroadenoma tissues that were closest representations of ‘cancer control’ breast tissues were used to know the source of the protein. Methylene blue was used to map the sentinel lymph node in axilla region of breast, followed by its excision. Histopathological analysis was done to annotate the tissue phenotype either SLNM + or SLNM- or benign tumor.

Proteins from sentinel lymph node tissues were isolated, quantified, digested with trypsin and labelled with different isobaric tags combined into one sample mixture for identification and quantification by LC–MS/MS analysis. Isobaric tags for relative and absolute quantitation (iTRAQ) technology relies on the quantitation of low molecular mass reporter ion groups released from isobaric tags that are covalently bind to primary amines of tryptic peptides that need to be quantified via amine labelling. The final experiment so designed was to enable: (i) six phenotypic protein profile comparisons, (ii) intra-experimental normalization and (iii) understand the cellular source of the protein. While the Up-regulated proteins in SLNM + , are related to tumorogenesis, cell proliferation, motility, cell survival, progression and anti-apoptosis, the up-regulated proteins in SLNM- are involved in cell motililty suppression and influenzing decreased cell growth. Expression of five proteins caveolin-1, collagen α1, desmin, fibrillin-1, and microfibrillar associated glycoprotein 4 were validated and were found to be consistent with the discovery phase results. The functions of these proteins in the context of understanding sentinel lymph node metastasis are: (a) Caveolin-1 (Cav-1), a 22 kDa small oligomeric scaffolding protein encoded by CAV1 gene is a major structural protein of membranes called caveolae and plays very crucial role in many cellular processes, including endocytosis, receptor internalization, ECM organization, lipid transport, signal transduction^86,87^; (b) Collagen αtype1 (COL1A1), a 138 kDa protein encoded by the COL1A1 gene is a most abundant protein of extracellular matrix forms a characteristic triple helix structure of three polypeptide chains, and contributes to the integrity, elasticity and strength of body's connective tissues, entrapment, local storage and delivery of growth factors and cytokines and therefore plays an important role during organ development, wound healing and tissue repair^88,89^; (c) Desmin (DES), a 53 kDa protein encoded by DES gene is a muscle-specific protein and a key subunit of the intermediate filament in cardiac, skeletal and smooth muscles, and plays an essential role in maintaining extracellular matrix interactions, cytoarchitecture, structural integrity and function of muscles by forming three dimensional scaffold across sarcomeres of smooth muscles^90,91^; (d) Fibrillin-1 (FBN1), a 312 kDa protein encoded by FBN1 gene, is a large cysteine rich glycoprotein produced by fibroblasts, and is the principal structural component of extracellular matrix forming microfibrils in the connective-tissue, and Interacting with other components of the extracellular matrix (ECM), this ubiquitous glycoprotein exert pivotal roles in tissue development, homeostasis and repair. In addition to mechanical support, FBN networks also exhibit regulatory activities on growth factor signalling, ECM formation, cell behaviour and the immune response^92,93^; and (e) Microfibrillar-associated glycoprotein 4 (MFAP4), a 36 kDa protein encoded by MFAP4 gene is an extracellular matrix protein that plays major role in elastin fiber formation and is associated with ECM remodeling processes during vascular injury, and interacts with other ECM proteins such as FBN1 that provides cell adhesion, intercellular interactions and the assembly and/or maintenance of elastic fibres^94,95^.

**Figure 7:**
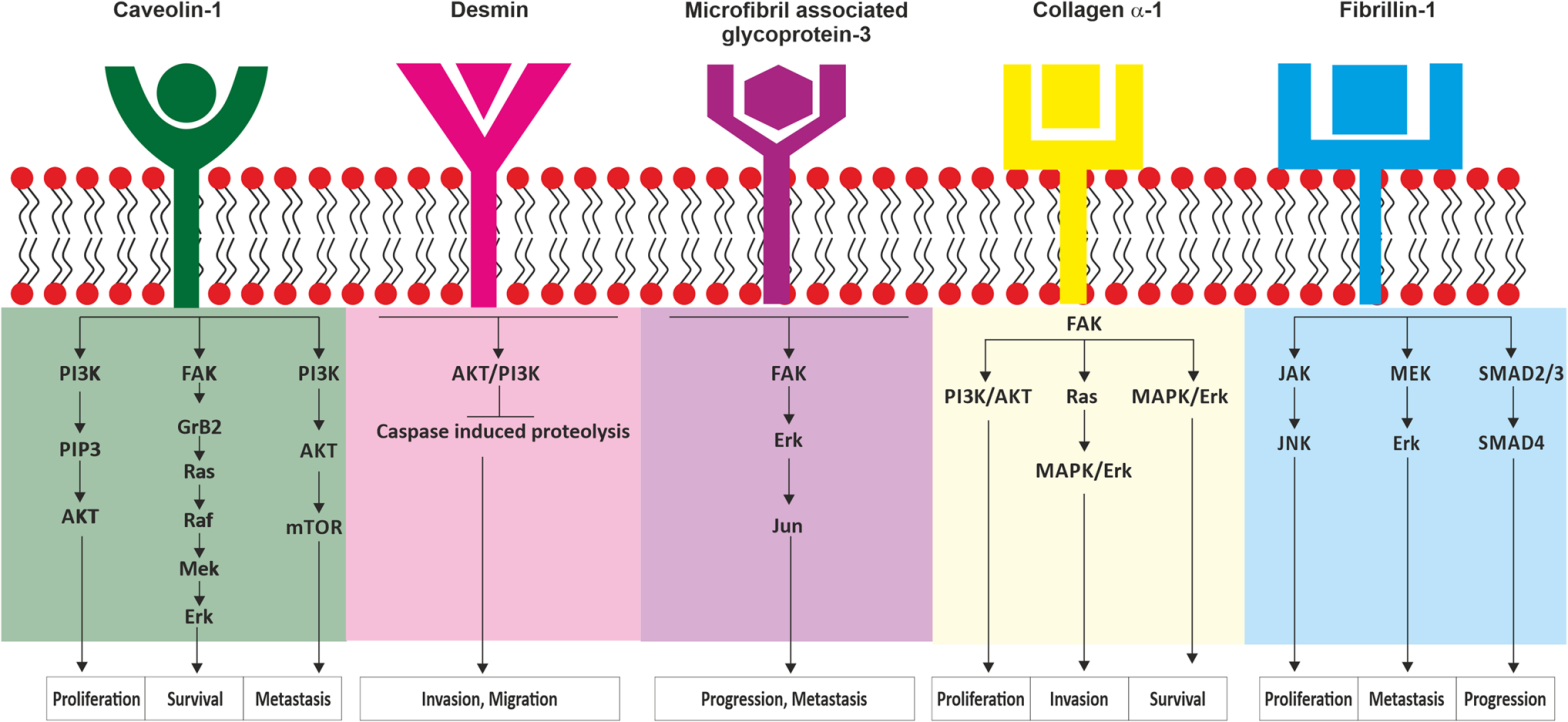
Diagrammatic representation showing oncogenic signalling pathways in breast cancer. Abbreviations: PI3K, Phosphatidylinositol 3-kinase; PIP3, Phosphatidylinositol (3,4,5)-trisphosphate; AKT, Protein kinase B; FAK, Focal adhesion kinase; GrB2, Growth factor receptor-bound protein 2; Ras, Rat sarcoma; Raf, Rapidly accelerated fibrosarcoma; Mek, Mitogen-activated protein kinase kinase; Erk, Extracellular signal-regulated kinase; mTOR, mechanistic target of rapamycin; PDK2, Pyruvate dehydrogenase kinase 2; Bcl2, B-cell lymphoma 2; NFkB, Nuclear factor kappa-light-chain-enhancer of activated B cells; JAK, Janus kinase; JNK, Jun N-terminal Kinase; SMAD, Small mothers against decapentaplegic.

Label based differential proteomic experiments, Pathway analysis, Gene Ontology studies, and ELISA experiments clearly establish the role of extracellular matrix proteins in sentinel lymph node metastasis. Extracellular matrices (ECMs) are highly specialized and dynamic three‐dimensional (3D) scaffolds into which cells reside in tissues and its principal components are collagens, glycoproteins, and proteoglycans^96^. Upon physiological and pathological triggers, ECM-degrading enzymes, matrikines, are released to remodel the ECM, to re-establish an appropriate functional meshwork and maintain cellular homeostasis^97,98^. But in metastasis, ECM remodeling is hijacked and there is perturbation and degradation in ECM architecture by matrix metalloproteinases^99,100^. Due to ECM degradation there is loss of endothelial integrity allowing cancer cells to escape from primary tumor to other tissues including lymph nodes^101^. During this process of metastasis, cancer cells undergo epithelial-to-mesenchymal transition (EMT), which can be induced by increased deposition of ECM proteins^102^. This action alters the phenotypic properties of cells and affects their propensity to escape primary tumor and cause metastasis^103^. In addition, an increased regulation of ECM proteins through recruitment and activation of cancer-associated fibroblasts (CAF) results in activation of biophysical and biochemical oncogenic signalling pathways^104^. The oncogenic signalling pathways of the identified extra cellular matrix proteins are (a) caveolin-1: PI3K/AKT and Ras/Raf signaling through the ERK; (b) Desmin: PI3K/AKT through caspase; (c) microfibril associated glycoprotein 4: ERK/MMP signalling through FAK and c-Jun; (d) collagen α-1: FAK signalling through PI3K/AKT and MAPK/ERK, and (e) fibrillin 1: SMAD2/3/4 and MEK pathway through ERK; which induces cell proliferation, survival, motility, angiogenesis, hypoxia, cancer stem cell activity, epithelial to mesenchymal transition and eventually lymph node metastasis^105–109^. Caveolin-1, Desmin and Collagen α-1 which mediate their functions through PI3K/Akt signalling which is represented along side ECM-receptor interaction in the pathway analysis. The overview of this detailed analysis is pictorially represented in Fig. 7. Therefore, it is this interplay between the up-regulated extra cellular matrix proteins, active growth factors of cancer cells, fibroblasts and signalling pathways, which together promote lymph node metastasis.

Caveolin-1, Desmin, microfibril associated glycoprotein 4, fibrillin 1 and collagenα-1 have been identified as potential biomarkers that can discriminate metastatic from non-metastatic sentinel lymph nodes in early breast cancer. To understand their ability to differentiate the two clinical phenotypes AUCs were plotted based on the ELISA concentrations (Supplementary Fig. 2). The areas were estimated to be 0.81 and 1.0 for these five proteins. Diagnostic parameters of sensitivity, specificity, positive predictive value and Negative predictive values are over 80% for each making them fairly accurate as a translational tool (Table 4). Caveolin-1 and Desmin with cut-off values of 17.4 and 28.5 ng/μg of tissue protein respectively, in particular are promising candidates with 100% values for all the diagnostic parameters. Since both the sensitivity and specificity measures are independent of prevalence rate, the practical utility of these two markers need to be validated among the population with varying prevalence rates.

**Table 4:** Diagnostic parameters of identified proteins to differentiate SLNM + from SLNM- in early breast cancer.

Protein	Cut off values (ng/µg of total protein)	Statistical Parameters							
		AUC (95% CI: LL- UL)	Sensitivity (%)	Specificity (%)	LR +	LR-	PPV (%)	NPV (%)	Correctly classified (%)
Collagen alpha 1	≥ 5.98	0.98 (0.86–1.00)	92.31	91.67	11.07	0.08	92.30	91.66	92.00
Fibrillin-1	≥ 5.17	0.81 (0.59–0.93)	84.62	83.33	5.07	0.18	84.61	81.81	84.00
Microfibril associated glycoprotein 4	≥ 254.3	0.96 (0.79–0.99)	84.62	91.67	10.15	0.16	91.66	84.61	88.00
Caveolin-1	≥ 17.40	1.0 (0.86–1.00)	100	100	NA	NA	100	100	100
Desmin	≥ 28.50	1.0 (0.8–1.00)	100	100	NA	NA	100	100	100

## Conclusion

iTRAQ based proteomic experiment is an ideal platform for comparative protein profiling in identification of potential biomarker candidates for sentinel lymph node metastasis in early breast cancer. Extra cellular matrix proteins caveolin-1, collagen α-1, desmin, fibrillin-1, and microfibrillar associated glycoprotein 4, have been identified as potential biomarkers that can differentiate the two metastatic states of sentinel lymph nodes. Each of these by themselves or as a collective panel offers translational scope for the design of ‘on-table’ diagnostics to flag sentinel lymph node metastasis in early breast cancer.

## Supplementary Information

Supplementary Information 1.

Supplementary Information 2.

Supplementary Information 3.

Supplementary Information 4.

## Data Availability

The mass spectrometry proteomics data have been deposited to the ProteomeXchange^110^ Consortium via the PRIDE partner repository with the dataset identifier **PXD027668.**

## References

[1] Wu H (2020). Isobaric tags for relative and absolute quantitation in proteomic analysis of potential biomarkers in invasive cancer, ductal carcinoma in situ, and mammary fibroadenoma. Front. Oncol..

[2] IARC. New Global Cancer Data: GLOBOCAN 2020. *Int. Agency Res. Cancer* **6** (2020).

[3] WHO. Globocan 2020. *International Agency for research* vol. 419 3–4 https://ascopost.com/news/december-2020/globocan-2020-database-provides-latest-global-data-on-cancer-burden-cancer-deaths/#:~:text=Female breast cancer has now, with 685%2C000 deaths in 2020. (2020).

[4] Sofi N, Jain M, Kapil U, Yadav C (2019). Epidemiological characteristics of breast cancer patients attending a tertiary health-care institute in the National Capital Territory of India. J. Cancer Res. Ther..

[5] Heer E (2020). Global burden and trends in premenopausal and postmenopausal breast cancer: A population-based study. Lancet Glob. Heal..

[6] Schwartz RS, Erban JK (2017). Timing of metastasis in breast cancer. N. Engl. J. Med..

[7] Bray F (2018). Global cancer statistics 2018: GLOBOCAN estimates of incidence and mortality worldwide for 36 cancers in 185 countries. CA. Cancer J. Clin..

[8] Weledji EP, Tambe J (2018). Breast cancer detection and screening. Med. Clin. Rev..

[9] Qiu Y (2019). A multiple breast cancer stem cell model to predict recurrence of T1–3, N0 breast cancer. BMC Cancer.

[10] Brenot-Rossi I (2003). Nonvisualization of axillary sentinel node during lymphoscintigraphy: Is there a pathologic significance in breast cancer?. J. Nucl. Med..

[11] Gradishar WJ (2018). Clinical practice guidelines in oncology. JNCCN J. Natl. Compr. Cancer Netw..

[12] Kelley MC, Hansen N, McMasters KM (2004). Lymphatic mapping and sentinel lymphadenectomy for breast cancer. Am. J. Surg..

[13] Krag DN, Weaver DL, Alex JC, Fairbank JT (1993). Surgical resection and radiolocalization of the sentinel lymph node in breast cancer using a gamma probe. Surg. Oncol..

[14] Noguchi M (1997). The role of axillary lymph node dissection in breast cancer management. Breast Cancer.

[15] Erb KM, Julian TB (2009). Completion of axillary dissection for a positive sentinel node: Necessary or not?. Curr. Oncol. Rep..

[16] Borgstein PJ (1998). Sentinel lymph node biopsy in breast cancer: Guidelines and pitfalls of lymphoscintigraphy and gamma probe detection. J. Am. Coll. Surg..

[17] van de Vijver MJ (2002). A gene-expression signature as a predictor of survival in breast cancer. N. Engl. J. Med..

[18] Lorusso G, Rüegg C (2012). New insights into the mechanisms of organ-specific breast cancer metastasis. Semin. Cancer Biol..

[19] Fry SA, Sinclair J, Timms JF, Leathem AJ, Dwek MV (2013). A targeted glycoproteomic approach identifies cadherin-5 as a novel biomarker of metastatic breast cancer. Cancer Lett..

[20] Zeng L (2017). Identification of nucleobindin-2 as a potential biomarker for breast cancer metastasis using iTRAQ-based quantitative proteomic analysis. J. Cancer..

[21] Mokhtar M (2016). Triple assessment of sentinel lymph node metastasis in early breast cancer using preoperative CTLG, intraoperative fluorescence navigation and OSNA. Breast Cancer.

[22] Zhao QL (2018). Elastosonography and two-dimensional ultrasonography in diagnosis of axillary lymph node metastasis in breast cancer. Clin. Radiol..

[23] Solon JG, Power C, Al-Azawi D, Duke D, Hill ADK (2012). Ultrasound-guided core biopsy: An effective method of detecting axillary nodal metastases. J. Am. Coll. Surg..

[24] Nandu VV, Chaudhari MS (2017). Efficacy of sentinel lymph node biopsy in detecting axillary metastasis in breast cancer using methylene blue. Indian J. Surg. Oncol..

[25] Harada T, Tanigawa N, Matsuki M, Nohara T, Narabayashi I (2007). Evaluation of lymph node metastases of breast cancer using ultrasmall superparamagnetic iron oxide-enhanced magnetic resonance imaging. Eur. J. Radiol..

[26] Roumen RMH, Valkenburg JGM, Geuskens LM (1997). Lymphoscintigraphy and feasibility of sentinel node biopsy in 83 patients with primary breast cancer. Eur. J. Surg. Oncol..

[27] Gupta AK (2019). Cerebrospinal fluid proteomics for identification of α2-macroglobulin as a potential biomarker to monitor pharmacological therapeutic efficacy in dopamine dictated disease states of Parkinson’s disease and schizophrenia. Neuropsychiatr. Dis. Treat..

[28] Pokhriyal R, Hariprasad R, Kumar L, Hariprasad G (2019). Chemotherapy resistance in advanced ovarian cancer patients. Biomark. Cancer..

[29] Pathania S (2020). Proteomics of sentinel lymph nodes in early breast cancer for identification of thymidylate synthase as a potential biomarker to flag metastasis: A preliminary study. Cancer Manag. Res..

[30] Kataria K, Srivastava A, Qaiser D (2016). What Is a false negative sentinel node biopsy: Definition, reasons and ways to minimize it?. Indian J. Surg..

[31] Orsburn BC (2021). Proteome discoverer-a community enhanced data processing suite for protein informatics. Proteomes.

[32] Huang DW, Sherman BT, Lempicki RA (2009). Systematic and integrative analysis of large gene lists using DAVID bioinformatics resources. Nat. Protoc..

[33] Pang Z (2021). MetaboAnalyst 5.0: Narrowing the gap between raw spectra and functional insights. Nucleic Acids Res..

[34] Kamradt MC, Chen F, Cryns VL (2001). The small heat shock protein αB-crystallin negatively regulates cytochrome c- and caspase-8-dependent activation of caspase-3 by inhibiting its autoproteolytic maturation. J. Biol. Chem..

[35] Mao YW, Liu JP, Xiang H, Li DWC (2004). Human αA- and αB-crystallins bind to Bax and Bcl-Xs to sequester their translocation during staurosporine-induced apoptosis. Cell Death Differ..

[36] Liu S (2014). As a novel p53 direct target, bidirectional gene HspB2/αB-crystallin regulates the ROS level and Warburg effect. Biochim. Biophys. Acta - Gene Regul. Mech..

[37] Shi C (2014). Alpha B-crystallin correlates with poor survival in colorectal cancer. Int. J. Clin. Exp. Pathol..

[38] Mellone M (2017). Induction of fibroblast senescence generates a non-fibrogenic myofibroblast phenotype that differentially impacts on cancer prognosis. Aging (Albany NY)..

[39] Hanley CJ (2016). A subset of myofibroblastic cancer-associated fibroblasts regulate collagen fiber elongation, which is prognostic in multiple cancers. Oncotarget.

[40] Li Y (2015). miR-181a-5p inhibits cancer cell migration and angiogenesis via downregulation of matrix metalloproteinase-14. Cancer Res..

[41] Gonzalez-Avila G (2019). Matrix metalloproteinases participation in the metastatic process and their diagnostic and therapeutic applications in cancer. Crit. Rev. Oncol. Hematol..

[42] Nieto MA, Huang RYYJ, Jackson RAA, Thiery JPP (2016). Emt: 2016. Cell.

[43] Ju Q (2019). Identification of a miRNA-mRNA network associated with lymph node metastasis in colorectal cancer. Oncol. Lett..

[44] Pike LJ (2005). Growth factor receptors, lipid rafts and caveolae: An evolving story. Biochim. Biophys. Acta – Mol. Cell Res..

[45] Badana AK (2018). Lipid rafts disruption induces apoptosis by attenuating expression of LRP6 and survivin in triple negative breast cancer. Biomed. Pharmacother..

[46] Wang R (2014). Caveolin-1 functions as a key regulator of 17β-estradiol-mediated autophagy and apoptosis in BT474 breast cancer cells. Int. J. Mol. Med..

[47] Bailey KM, Liu J (2008). Caveolin-1 up-regulation during epithelial to mesenchymal transition is mediated by focal adhesion kinase. J. Biol. Chem..

[48] Gai X, Lu Z, Tu K, Liang Z, Zheng X (2014). Caveolin-1 is up-regulated by GLI1 and contributes to GLI1-driven EMT in hepatocellular carcinoma. PLoS One..

[49] Joglekar M, Elbazanti WO, Weitzman MD, Lehman HL, van Golen KL (2017). Erratum to: Caveolin-1 mediates inflammatory breast cancer cell invasion via the Akt1 pathway and RhoC GTPase: RhoC and caveolin -1 in inflammatory breast cancer. J. Cell. Biochem..

[50] Li S (2019). Shear stress promotes anoikis resistance of cancer cells via caveolin-1-dependent extrinsic and intrinsic apoptotic pathways. J. Cell. Physiol..

[51] Wang K, Zhu X, Chen Y, Yin Y, Ma T (2018). Tubeimoside V sensitizes human triple negative breast cancer MDA-MB-231 cells to anoikis via regulating caveolin-1-related signaling pathways. Arch. Biochem. Biophys..

[52] Kim SH (2014). Role of secreted type I collagen derived from stromal cells in two breast cancer cell lines. Oncol. Lett..

[53] Liu J (2018). Collagen 1A1 (COL1A1) promotes metastasis of breast cancer and is a potential therapeutic target. Discov. Med..

[54] De Wever O, Mareel M (2003). Role of tissue stroma in cancer cell invasion. Journal of Pathology..

[55] Zilberberg L (2012). Specificity of latent TGF-β binding protein (LTBP) incorporation into matrix: Role of fibrillins and fibronectin. J. Cell. Physiol..

[56] Fuxe J, Vincent T, De Herreros AG (2010). Transcriptional crosstalk between TGFβ and stem cell pathways in tumor cell invasion: Role of EMT promoting Smad complexes. Cell Cycle.

[57] Chaudhury A (2010). TGF-Β-mediated phosphorylation of hnRNP E1 induces EMT via transcript-selective translational induction of Dab2 and ILEI. Nat. Cell Biol..

[58] Liu K (2017). Glyceraldehyde-3-phosphate dehydrogenase promotes cancer growth and metastasis through upregulation of SNAIL expression. Int. J. Oncol..

[59] Calderwood SK, Gong J (2016). Heat shock proteins promote cancer: It’s a protection racket. Trends Biochem. Sci..

[60] Tsutsumi S, Beebe K, Neckers L (2009). Impact of heat-shock protein 90 on cancer metastasis. Future Oncol..

[61] Shiota M (2013). Hsp27 regulates epithelial mesenchymal transition, metastasis, and circulating tumor cells in prostate cancer. Cancer Res..

[62] Kim WH, Lee BL, Kim DK, Kleinman HK (1999). Laminin-1-adherent cancer cells show increased proliferation and decreased apoptosis in vivo. Anticancer Res..

[63] Givant-Horwitz V, Davidson B, Reich R (2005). Laminin-induced signaling in tumor cells. Cancer Lett..

[64] Wu JB (2014). Monoamine oxidase A mediates prostate tumorigenesis and cancer metastasis. J. Clin. Invest..

[65] Albig AR, Becenti DJ, Roy TG, Schiemann WP (2008). Microfibril-associate glycoprotein-2 (MAGP-2) promotes angiogenic cell sprouting by blocking notch signaling in endothelial cells. Microvasc. Res..

[66] Yang J (2019). Integrated analysis of microfibrillar-associated proteins reveals MFAP4 as a novel biomarker in human cancers. Epigenomics.

[67] Huang Y, Arora P, McCulloch CA, Vogel WF (2009). The collagen receptor DDR1 regulates cell spreading and motility by associating with myosin IIA. J. Cell Sci..

[68] Arjonen A (2014). Mutant p53.associated myosin-X upregulation promotes breast cancer invasion and metastasis. J. Clin. Invest..

[69] Tirinato L (2017). An overview of lipid droplets in cancer and cancer stem cells. Stem Cells Int..

[70] Chen YF (2016). MicroRNA-211 enhances the oncogenicity of carcinogen-induced oral carcinoma by repressing TCF12 and increasing antioxidant activity. Cancer Res..

[71] Yu H (2008). Transgelin is a direct target of TGF-β/Smad3-dependent epithelial cell migration in lung fibrosis. FASEB J..

[72] Lin Y (2009). Association of the actin-binding protein transgelin with lymph node metastasis in human colorectal cancer. Neoplasia.

[73] Wu X, Dong L, Zhang R, Ying K, Shen H (2014). Transgelin overexpression in lung adenocarcinoma is associated with tumor progression. Int. J. Mol. Med..

[74] Borensztajn K, Peppelenbosch MP, Spek CA (2010). Coagulation Factor Xa inhibits cancer cell migration via LIMK1-mediated cofilin inactivation. Thromb. Res..

[75] Zhou ZJ (2014). Hnrnpab induces epithelial-mesenchymal transition and promotes metastasis of hepatocellular carcinoma by transcriptionally activating snail. Cancer Res..

[76] Stockley J (2014). The RNA-binding protein hnRNPA2 regulates β-catenin protein expression and is overexpressed in prostate cancer. RNA Biol..

[77] Deng J (2016). Effects of hnRNP A2/B1 knockdown on inhibition of glioblastoma cell invasion, growth and survival. Mol. Neurobiol..

[78] Tauler J, Zudaire E, Liu H, Shih J, Mulshine JL (2010). hnRNP A2/B1 modulates epithelial-mesenchymal transition in lung cancer cell lines. Cancer Res..

[79] Bianchi ME, Agresti A (2005). HMG proteins: Dynamic players in gene regulation and differentiation. Curr. Opin. Genet. Dev..

[80] Medrzycki M (2014). Histone H1.3 suppresses H19 noncoding RNA expression and cell growth of ovarian cancer cells. Cancer Res..

[81] Wang J (2019). Cathelicidin suppresses colon cancer metastasis via a P2RX7-dependent mechanism. Mol. Ther. - Oncolytics..

[82] Kang M (2013). miR-129-2 suppresses proliferation and migration of esophageal carcinoma cells through downregulation of SOX4 expression. Int. J. Mol. Med..

[83] Dransart E, Olofsson B, Cherfils J (2005). RhoGDIs revisited: Novel roles in Rho regulation. Traffic.

[84] Hooper JA (1975). Purification and properties of Bovine Thymosin. Ann. N. Y. Acad. Sci..

[85] Fan Y-z, Chang H, Ye Y, Liu J, Wang R (2006). Thymosin α1 suppresses proliferation and induces apoptosis in human leukemia cell lines. Peptides.

[86] Williams TM, Lisanti MP (2004). The caveolin proteins. Genome Biol..

[87] Xu Y (2017). Endocytosis and membrane receptor internalization: Implication of F-BAR protein Carom. Front. Biosci. - Landmark..

[88] Gajko-Galicka A (2002). Mutations in type I collagen genes resulting in osteogenesis imperfecta in humans. Acta Biochim. Pol..

[89] Gelse K, Pöschl E, Aigner T (2003). Collagens - Structure, function, and biosynthesis. Adv. Drug Deliv. Rev..

[90] Paulin D, Li Z (2004). Desmin: A major intermediate filament protein essential for the structural integrity and function of muscle. Exp. Cell Res..

[91] Shah SB (2004). Structural and functional roles of desmin in mouse skeletal muscle during passive deformation. Biophys. J..

[92] Sakai LY, Keene DR, Renard M, De Backer J (2016). FBN1: The disease-causing gene for Marfan syndrome and other genetic disorders. Gene.

[93] Schrenk S, Cenzi C, Bertalot T, Conconi MT, Di Liddo R (2018). Structural and functional failure of fibrillin-1 in human diseases (Review). Int. J. Mol. Med..

[94] Pilecki B (2016). Characterization of microfibrillar-associated protein 4 (MFAP4) as a tropoelastin- and fibrillin-binding protein involved in elastic fiber formation. J. Biol. Chem..

[95] Kielty CM (2017). Fell-Muir lecture: Fibrillin microfibrils: structural tensometers of elastic tissues?. Int. J. Exp. Pathol..

[96] Morgado FN, da Silva AVA, Porrozzi R (2020). Infectious diseases and the lymphoid extracellular matrix remodeling: A focus on conduit system. Cells.

[97] Fares J, Fares MY, Khachfe HH, Salhab HA, Fares Y (2020). Molecular principles of metastasis: A hallmark of cancer revisited. Signal Transduct. Target. Ther..

[98] Wiig H, Keskin D, Kalluri R (2010). Interaction between the extracellular matrix and lymphatics: Consequences for lymphangiogenesis and lymphatic function. Matrix Biol..

[99] Kaushik S, Pickup MW, Weaver VM (2016). From transformation to metastasis: Deconstructing the extracellular matrix in breast cancer. Cancer Metastasis Rev..

[100] Winkler J, Abisoye-Ogunniyan A, Metcalf KJ, Werb Z (2020). Concepts of extracellular matrix remodelling in tumour progression and metastasis. Nat. Commun..

[101] Ecker BL (2019). Age-related changes in HAPLN1 increase lymphatic permeability and affect routes of melanoma metastasis. Cancer Discov..

[102] Paszek MJ (2005). Tensional homeostasis and the malignant phenotype. Cancer Cell.

[103] Frantz C, Stewart KM, Weaver VM (2010). The extracellular matrix at a glance. J. Cell Sci..

[104] Erdogan B, Webb DJ (2017). Cancer-associated fibroblasts modulate growth factor signaling and extracellular matrix remodeling to regulate tumor metastasis. Biochem. Soc. Trans..

[105] Kortum RL (2014). Caveolin-1 is required for kinase suppressor of Ras 1 (KSR1)-mediated extracellular signal-regulated kinase 1/2 activation, H-Ras V12 -induced senescence, and transformation. Mol. Cell. Biol..

[106] Zhang H (2019). LAMB3 mediates apoptotic, proliferative, invasive, and metastatic behaviors in pancreatic cancer by regulating the PI3K/Akt signaling pathway. Cell Death Dis..

[107] Xia P, Liu Y, Cheng Z (2016). Signaling pathways in cardiac myocyte apoptosis. Biomed. Res. Int..

[108] Mecham RP, Gibson MA (2015). The microfibril-associated glycoproteins (MAGPs) and the microfibrillar niche. Matrix Biol..

[109] Xiong H (2008). Inhibition of JAK1, 2/STAT3 signaling induces apoptosis, cell cycle arrest, and reduces tumor cell invasion in colorectal cancer cells. Neoplasia.

[110] Deutsch EW (2020). The ProteomeXchange consortium in 2020: Enabling ‘big data’ approaches in proteomics. Nucleic Acids Res..

