# Supplementary figures and images for: iTRAQ proteomics of sentinel lymph nodes for identification of extracellular matrix proteins to flag metastasis in early breast cancer
Source: Sci Rep. 2022 May 22;12:8625. doi: 10.1038/s41598-022-12352-9 (PMC9124668; doi:10.1038/s41598-022-12352-9)

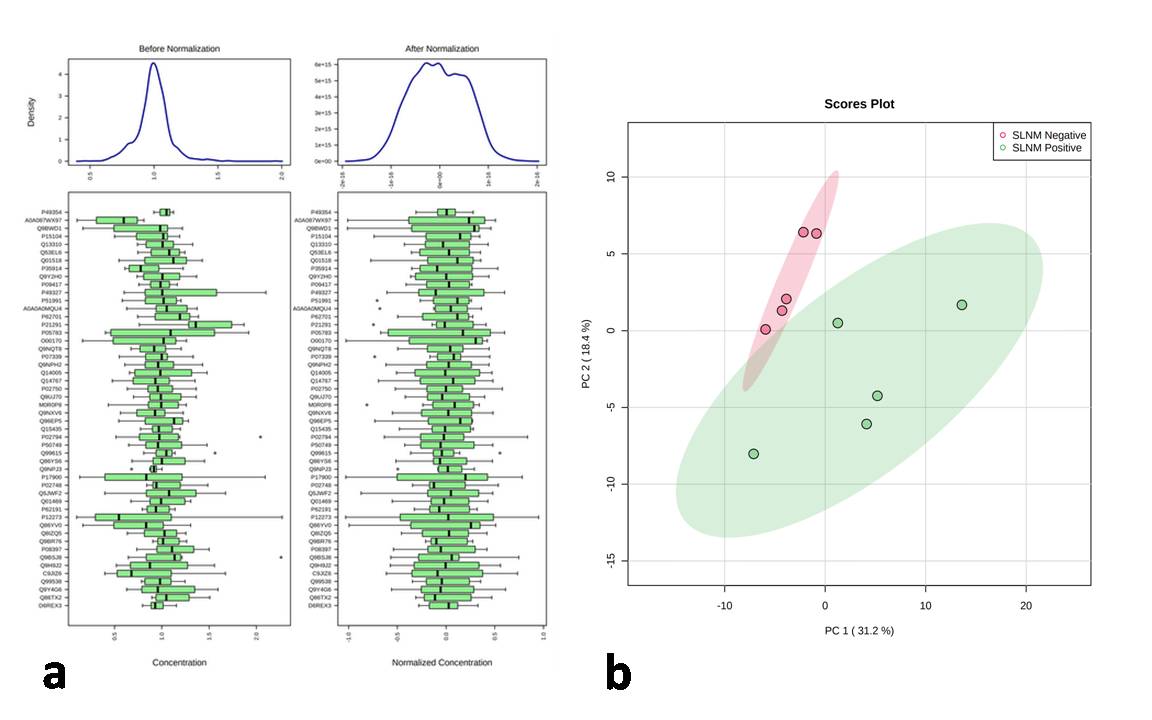

Supplement: Supplementary file 1 — Supplementary Information 1. [file 41598_2022_12352_MOESM1_ESM.jpg]

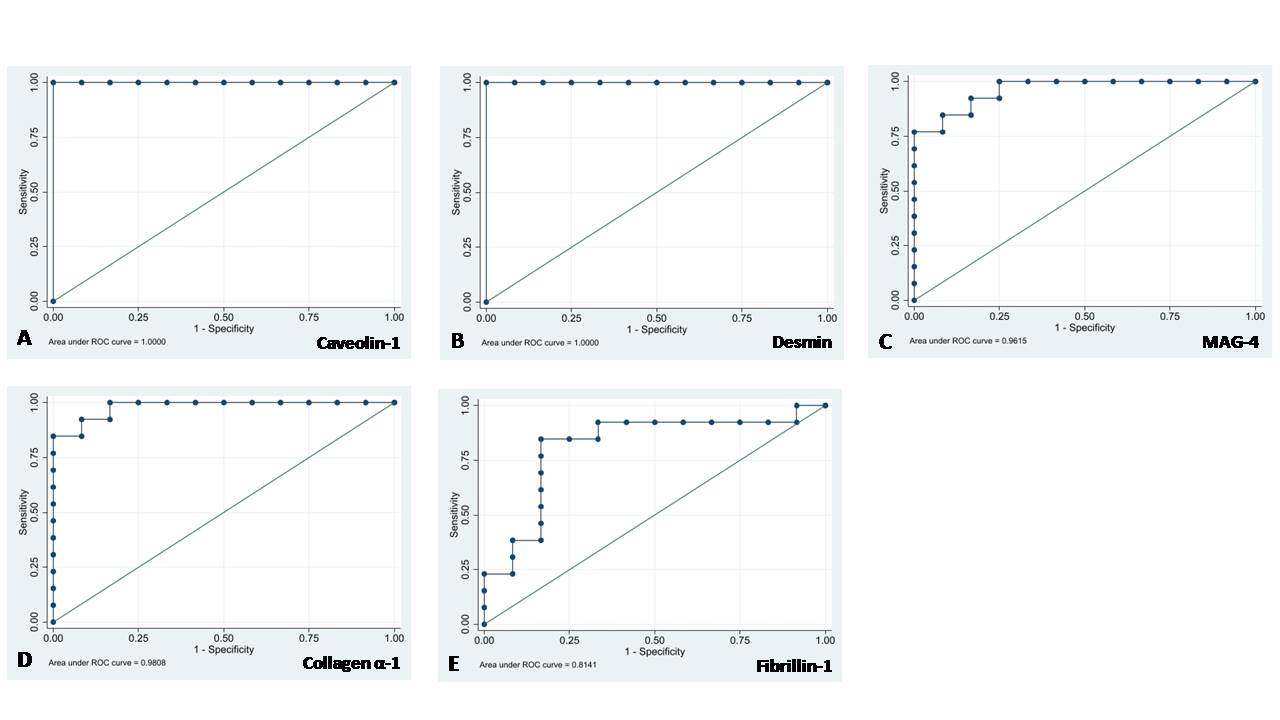

Supplement: Supplementary file 4 — Supplementary Information 4. [file 41598_2022_12352_MOESM4_ESM.jpg]
