# Supplementary material for: iTRAQ proteomics of sentinel lymph nodes for identification of extracellular matrix proteins to flag metastasis in early breast cancer
Source: Sci Rep. 2022 May 22;12:8625. doi: 10.1038/s41598-022-12352-9 (PMC9124668; doi:10.1038/s41598-022-12352-9)
Supplement: Supplementary file 2 — Supplementary Information 2. [file 41598_2022_12352_MOESM2_ESM.docx]

**Supplementary Figure 1.**

1. **Normalization of differentially expressed proteins.** Upper panel shows the density plots generated using all the 2398 proteins before and after normalization in the left and right panels, respectively. Lower panel shows boxplots of the top 50 representative proteins before and after normalization. **(B)** **2D Score plot to differentiate SLNM positive and SLNM negative groups.** Multivariate unsupervised Principal Component Analysis (PCA) of SLNM positive and SLNM negative groups. Principal Component 1 (PC1) and Principal Component 2 (PC2) are plotted in x and y axes, respectively, and together have 49.6% of variation. PC1 and PC2 display 95% confidence regions. Pink and green ovals designate the clustering regions of SLNM- and SLNM+ groups, respectively.

**Supplementary Figure 2.**

**Receiver Operating Characteristic (ROC) for cut-offs that best differentiate SLNM+ from SLNM-.**(a) caveolin-1, (b) desmin, (c) microfibrillar associated glycoprotein 4 (d) collagen α-1, and (e) fibrillin-1
