# Supplementary material for: iTRAQ proteomics of sentinel lymph nodes for identification of extracellular matrix proteins to flag metastasis in early breast cancer
Source: Sci Rep. 2022 May 22;12:8625. doi: 10.1038/s41598-022-12352-9 (PMC9124668; doi:10.1038/s41598-022-12352-9)
Supplement: Supplementary file 3 — Supplementary Information 3. [file 41598_2022_12352_MOESM3_ESM.docx]

| **Caveolin 1**  (ng/μg of total protein) | | **Desmin**  (ng/μg of total protein) | | **Microfibrillar associated glycoprotein 4**  (ng/μg of total protein) | | **Collagen alpha 1**  (ng/μg of total protein) | | **Fibrillin 1**  (ng/μg of total protein) | |
| --- | --- | --- | --- | --- | --- | --- | --- | --- | --- |
| **SLNM+** | **SLNM-** | **SLNM+** | **SLNM-** | **SLNM+** | **SLNM-** | **SLNM+** | **SLNM-** | **SLNM+** | **SLNM-** |
| 33.53 | 12.41 | 33.62 | 23.99 | 331.89 | 214.75 | 4.95 | 3.65 | 5.48 | 4.40 |
| 31.86 | 12.53 | 39.41 | 26.24 | 390.64 | 255.46 | 10.01 | 3.31 | 3.88 | 4.81 |
| 32.67 | 13.60 | 36.72 | 23.68 | 401.53 | 108.85 | 9.23 | 6.23 | 6.39 | 4.13 |
| 31.14 | 16.32 | 33.38 | 25.11 | 276.89 | 120.10 | 8.88 | 4.36 | 5.20 | 4.81 |
| 32.28 | 17.05 | 34.08 | 25.18 | 299.21 | 91.89 | 9.56 | 4.24 | 4.90 | 4.68 |
| 30.77 | 17.40 | 32.92 | 19.37 | 312.25 | 148.50 | 11.45 | 3.51 | 6.15 | 5.02 |
| 33.37 | 16.72 | 36.69 | 18.22 | 187.96 | 138.32 | 9.23 | 3.89 | 5.17 | 6.15 |
| 34.76 | 13.34 | 31.23 | 26.72 | 254.39 | 118.85 | 8.74 | 3.32 | 5.57 | 5.65 |
| 30.36 | 14.34 | 33.28 | 28.02 | 280.82 | 174.03 | 5.98 | 2.59 | 5.54 | 5.06 |
| 32.75 | 17.16 | 28.5 | 23.81 | 262.96 | 194.03 | 7.71 | 3.60 | 6.91 | 4.76 |
| 29.80 | 14.70 | 32.52 | 26.47 | 299.57 | 175.82 | 7.66 | 5.15 | 5.35 | 4.83 |
| 34.12 | 11.68 | 54.63 | 18.97 | 319.75 | 25.82 | 6.87 | 3.63 | 6.12 | 3.19 |
| 36.09 |  | 33.56 |  | 206.89 |  | 8.18 |  | 11.44 |  |

**Supplementary Table 1.** Concentrations of proteins in metastatic and Non-metastatic sentinel lymph nodes on ELISA
